# Financial Data Analysis and Application Based on Big Data Mining Technology

**DOI:** 10.1155/2022/6711470

**Published:** 2022-06-25

**Authors:** Jinfeng Cheng

**Affiliations:** School of Finance and Accounting, Henan Industry and Trade Vocational College, ZhengZhou 451191, China

## Abstract

We provide a brief overview of the connotation and characteristics of data mining technology in the era of big data, analyze the feasibility of data mining technology in business management from the economic and technical perspectives, and propose specific application suggestions according to the content and requirements of business management. This paper describes in detail the principles and steps of using the weighted plain Bayesian algorithm and the decision tree algorithm to analyze students' performance; firstly, we need to obtain the plain Bayesian analysis model of college students' learning literacy in physical education and the C4.5 graduation literacy analysis model, and then use certain rules to combine the weighted plain Bayesian algorithm and the decision tree algorithm to obtain the WNB-C4.5 college students' learning literacy analysis model. In addition, in the prediction of financial risks, the classification scheme can be used in the judgment of violation of regulations, but the most used classification scheme is the decision tree. Experiments show that the effectiveness of this scheme in data mining for financial companies is increased by 2% compared to the benchmark method.

## 1. Introduction

With the rapid development of Internet, cloud computing, and Internet of Things (IoT) technologies in recent years, modern society has gradually stepped into the era of informatization and data-oriented environment [1]. In the development of enterprises, production and operation activities will generate a lot of data and the trend of explosive growth [2]. Comprehensive retrieval, analysis, and application of data can lay a good foundation for the formulation of scientific decision-making, and data information has gradually become an important factor affecting the development capacity of enterprises [3, 4].

With the continuous development of data mining technology, researchers have been expanding data mining technology, which makes its application research fields become more and more extensive [5]. At present, a large number of data mining techniques are successfully applied in many fields such as medical and health care, national defense science and technology, education and teaching, enterprise applications, and communication industry, which are widely concerned by researchers [6].

For example, in the area of intelligent decision support system, few researchers [7, 8] researched and designed an intelligent decision support system based on data warehouse, OLAP, and data mining methods, and also researched and designed a new intelligent decision architecture framework. In terms of data warehouse applications, Liu et al. [9] researched and implemented a management system applicable to customer data analysis based on data warehousing and data mining techniques, and the combination of the two techniques reflects the advantages of analyzing historical data and is widely used in the mobile communication industry. Few researchers [10, 11] analyzed the intelligent financial decision support system of the ZT Group by combining three key mining techniques, namely association rules, fuzzy methods, and unstructured data mining techniques, and also adopted a function mapping approach to achieve improved efficiency of operations in response to the shortcomings of the above three techniques. Similar analysis of intelligent decision support system based on data mining technology has many other worthy examples [12].

With the wide application of data mining techniques, most universities also apply the techniques commonly used in data mining to their daily educational teaching activities. Cui and Yan [13] designed and implemented an efficient grade analysis system based on data mining. The system adopts the grade analysis method of data mining, which can quickly and efficiently uncover valuable potential information hidden in a large amount of grade data and help university academic staff to comprehensively analyze students' grades. In [14], data mining technology is applied to the university reader borrowing query and analysis management system, the association rule mining technique is studied in depth, and the classical Apriori algorithm is analyzed, while the Apriori algorithm is improved, thus improving the efficiency of the algorithm to a great extent.

In the metrology business processing, the traditional metrology business often fails to extract valuable data information quickly and effectively when dealing with huge data, which restricts the metrology business management decisions [15]. The use of data mining technology, by building data mining models and data warehouses can effectively handle the huge amount of data generated in metrology business, thus reducing the errors in metrology business to within the standard range and improving the efficiency of metrology business. In [16], the application of data mining technology in WAP business operation is described. By analyzing and comparing the advantages and disadvantages characteristics of various data mining methods, the association algorithm is finally selected to mine the access logs generated by WAP, and some practical optimization solutions are proposed for the performance of data warehouse and data mining.

The deep application of data mining techniques is also widely involved in medical applications. Xu et al. [17] improved the Apriori algorithm by analyzing classical association rule mining algorithms such as AIS algorithm, FP-Growth, and Apriori algorithm, and proposed an array-based mining association rule DRA algorithm, which greatly improves the operation efficiency because the DRA algorithm does not need to generate candidate sets. In [18], a design idea of a data mining system for TCM cases with gastric pain was proposed, and the application of classical association algorithm in TCM cases with gastric pain was effectively verified by mining the medication pattern in 1221 cases for treating a certain disease using the Apriori algorithm.

From the above literature review, it is easy to find that data mining technology is now involved in almost every aspect of people's daily production life, and it is also used in intelligent decision support systems, higher education institutions, metrology business processing, mobile dream network data business, medical business, and other fields with increasing maturity [19, 20]. Based on the respective characteristics of classical Apriori association rule, clustering algorithm, and decision tree algorithm in data mining technology, we decided to use the above three data mining algorithms to realize the analysis of the enterprise's financial data so as to uncover the potential value information in the enterprise's financial data and provide a reliable decision basis for the enterprise's leadership.

## 2. System Business Requirements Analysis

### 2.1. System Process Analysis

**Figure 1:**
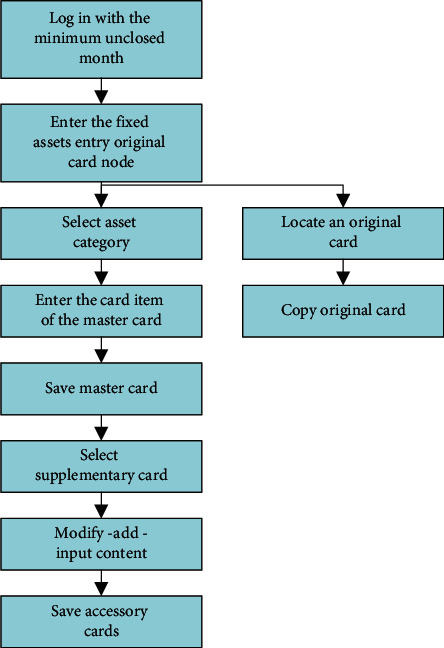
Original financial card management process.

The system process analysis mainly describes the execution process of a core business in the main functional modules of the system. Since the financial management system has more functions and the accompanying business process is also relatively large, in view of this, this chapter will focus on analyzing the original financial card management process in the financial management system. The original financial fixed assets card management specification process is shown in Figure 1.

  Step 1: login to the system with the minimum open month.
  Step 2: enter the “Fixed Assets” management operation and enter the original card node; locate an original card at the same time and copy the original card operation.
  Step 3: select the fixed assets category.
  Step 4: enter the items of this fixed assets master card.
  Step 5: save the card.
  Step 6: select the attached card.
  Step 7: make changes to the selected supplementary card, add another supplementary card, and enter the contents again.
  Step 8: save the card.

Through the above eight steps, you can realize the original financial card data entry workflow.

**Figure 2:**
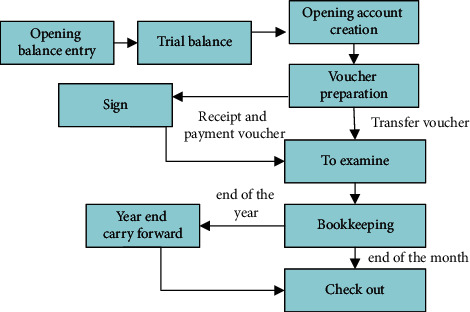
General ledger management process.

The general ledger of enterprise assets is an accounting of enterprise fixed assets according to certain classification standards in a certain period of all economic operations, the original value of the assets, accumulated depreciation, net value (provision for impairment, net) in a three-column format of debit, credit, and balance of the summary to reflect the changes in their value of the pages of the account. The flow chart of general ledger management is shown in Figure 2.

The general ledger process includes the initial balance entry and after the trial balance, the initial accounts can be created. The general ledger manager can then create some account vouchers and documents based on the initial accounts, and by signing and stamping on the postpayment vouchers, eventually form transfer vouchers for year-end account review and audit role, and finally form bookkeeping methods for year-end transfer.

## 3. Data Mining Technology in the Era of Big Data

**Figure 3:**
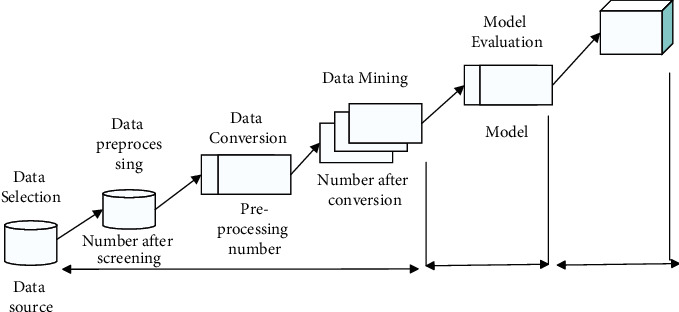
Flow of big data mining.

Big data mining technology is an important constituent element of knowledge discovery to analyze data with computer algorithms. In a large number of databases, the required data is obtained, and the data is appropriately transformed, mined, and utilized to obtain valuable information. Generally speaking, the object of big data mining is basically structured, semistructured, or other structured data. The process of data mining is mainly data selection ⟶ data mining ⟶ data analysis (see Figure 3).

## 4. Financial Analysis Method Based on Weighted Multiple Random Decision Trees

**Figure 4:**
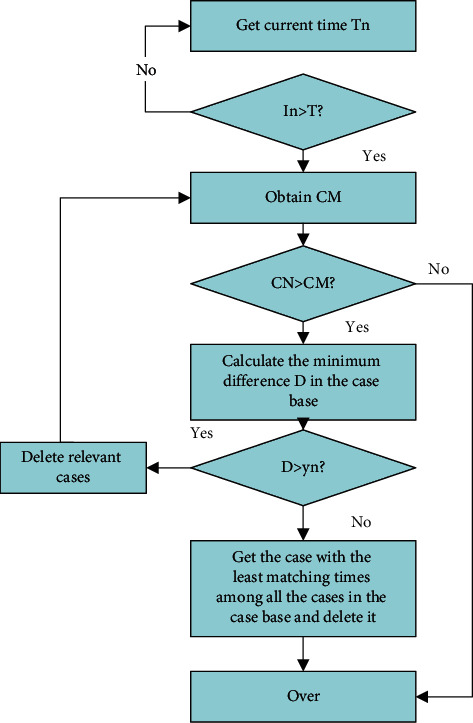
Case deletion process.

The classification problem of financial data is completed by adding a random decision tree scheme to the model, as shown in Figure 4.

The criticality of the attributes in the financial data warehouse varies under different mining objectives, so the criticality of each attribute should be analyzed quantitatively when establishing the decision tree. The current schemes that are often used to confirm the criticality of attributes are the discriminant matrix-based scheme and the information entropy-based scheme. In this study, we use the discriminant matrix scheme to evaluate the importance of attributes. In addition, since financial data are highly specialized, it is not possible to reflect the actual importance of an attribute by relying only on the discriminant matrix, so this project adds artificial weights to modify and intervene in the discriminant matrix to make the calculation of attribute weights more accurate [21, 22].

### 4.1. Defining the Resolution Matrix

A diagonal matrix of |*u*| × |*u*|. Each of these terms is defined as

(1) $$\begin{array}{c} C_{ij}=\left\{\begin{array}{c} \left\{\alpha \in A|\alpha \left(x_{i}\right)\neq \alpha \left(x_{j}\right)\right\}d\left(x_{i}\right)\neq d\left(x_{j}\right),d\left(x\right)\in D\\ \phi \;d\left(x_{i}\right)=d\left(x_{j}\right),d\left(x\right)\in D \end{array}\right.. \end{array}$$

The number of occurrences and the importance of the attributes in the discrimination matrix are positively correlated; and the shorter the data item with the attribute present, the more critical the attribute.

### 4.2. Calculating Attribute Weights for Financial Data

Initialize all *a*_*i*_ ∈ *A* such that *w*(*a*_*i*_)=0.

For each term of the diagonal matrix in the resolution matrix *C*_*jk*_ calculate

(2) $$\begin{array}{c} w\left(a_{i}\right)=w\left(a_{i}\right)+\left|C_{jk}\right|,\\ a_{i}\in C_{jk},0<k<j=\left|U\right|. \end{array}$$

In the above equation, |*A*| is the base of all attributes and |*C*_*jk*_| is the base of the discrimination matrix *C*_*jk*_.

After the system presents the weights, it is possible to manually correct the weights in the system, so it is necessary to add the correction coefficients *w*^*I*^(*a*_*i*_), −1 < =*w*^*I*^(*a*_*i*_) < =1, if you want to increase the weight of *a*_*i*_ by setting *w*^*I*^(*a*_*i*_) to a positive value, and the opposite by setting it to a negative value, then the weight of attribute *a*_*i*_ is *W*_*ai*_=*w*(*a*_*i*_)+*w*^*I*^(*a*_*i*_).

## 5. Experimental Validation

This validation data are derived from the financial statistics of more than 1400 company customers who have worked with a commercial bank, and the period of validation data are uniform from 2013 to 2016. The financial information data tables are divided into attributes based on the bank's transaction database, so the financial information data tables provided by the bank can be transformed into 24 attributes that clearly show the financial situation of the company, as presented in Table 1.

Due to the actual situation of the company in 2017 and the indicators related to the company, experts in finance classify the company risk into four categories: large, large, small, and small. In this case, companies with high risk are those that will go bankrupt from 2015 to 2017; companies with high risk are those that will default; companies with low risk are those that will not default but their financial situation will deteriorate, and companies with low risk have a normal financial situation and will not default. The results of the study showed that the best way to apply the decision is to build 10 random decision trees. Therefore, in this study, a total of 10 randomized decision trees were built from the analyzed data because the decision trees were built in a randomized manner, and a total of 5 trials were conducted to verify the stability of the decision trees [23, 24].

**Figure 5:**
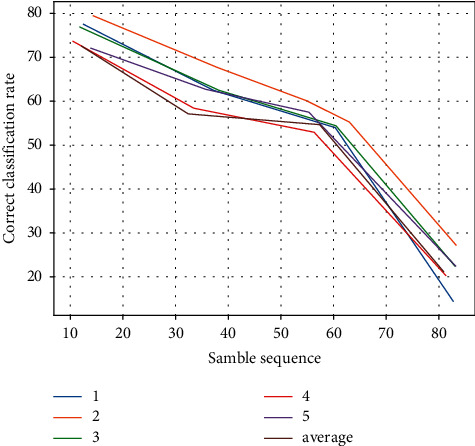
Comparison of the correct classification rate of multiple randomized decision trees.

The remaining 300 data are test data. The training data were used to build a random decision tree, and the completed decision tree was tested using the test data to finally document the classification accuracy of the decision tree. The experimental results are presented in Table 2 and Figure 5.

The results of the experiment show that this randomized decision tree algorithm classifies companies with large risk, large risk, small risk, and small risk with improved classification accuracy, which has been determined by bank staff to be a practical reference for predicting bank risk. However, because of the small number of large risk data in the training dataset, this branch is not sufficiently trained, making the stochastic decision tree algorithm less accurate than the other branches for large risk classification [25].

**Figure 6:**
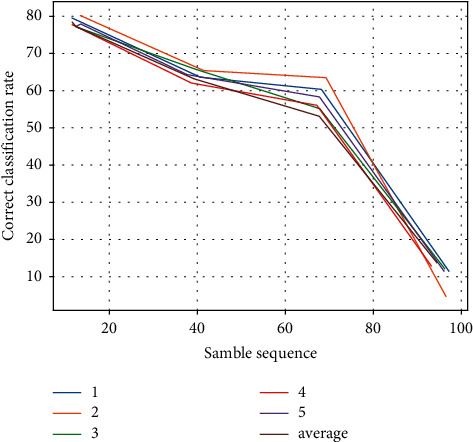
Comparison of the correct classification rate of C4.5 algorithm.

Each time, using the same training and validation data, the C4.5 algorithm is applied to classify the risk level, and the final results are presented in Table 3 and Figure 6.

**Figure 7:**
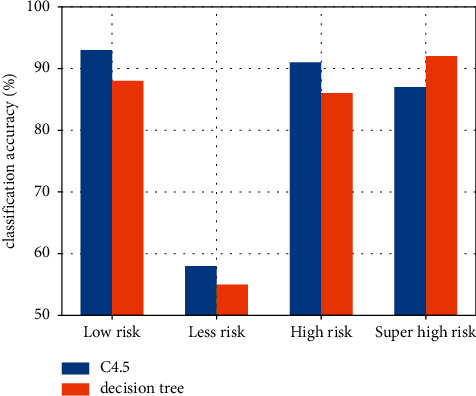
Comparison of the classification accuracy of the two algorithms.

The results of the experiments show that this randomized decision tree algorithm improves the classification accuracy for the risk level of large, risk level of large, risk level of small, and risk level of small by a considerable amount. Similarly, it can be seen that because the number of data with large risk level in the training dataset is relatively small, this class of branches is not trained sufficiently. The accuracy of the C4.5 algorithm is significantly lower for the risky branches compared to the other branches. This is shown in Figure 7.

From Figure 7, we can see that the accuracy of the randomized decision tree algorithm is higher than that of the C4.5 algorithm, which is about 10% higher.

**Figure 8:**
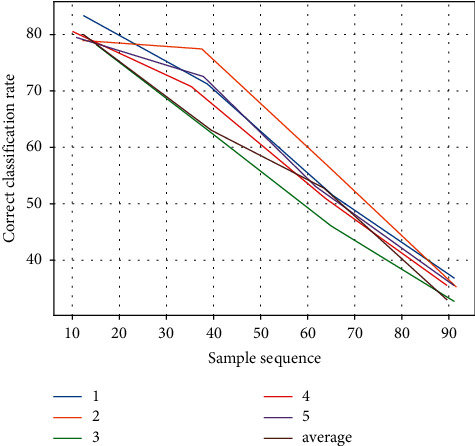
Comparison of the correct classification rate for stratified sampling of multiple random decision trees.

**Figure 9:**
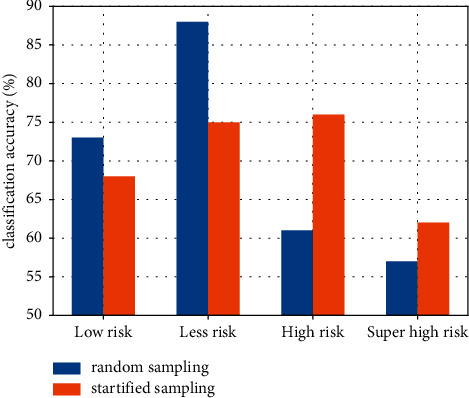
Comparison of the correct classification rate of random sampling and stratified sampling.

In order to improve the correct rate, 300 data with high risk level are added to the training data set because the training of high risk level is not sufficient. The number of training data with large risk is ensured by replacing the original random sampling with stratified sampling, in which the initial data are stratified by small, small, large, and large risk, and then random sampling is used for each stratum. The classification results using the stratified random sampling method are presented in Table 4 and Figures 8 and 9.

From the above figure, we can see that the correct rate of using the stratified sampling method with high risk is 10% higher than that of the random sampling method with high risk. The underlying reason is that 300 risky data are added to the training data set, which provides more samples for the stratified sampling. Therefore, the number of samples in the training data determines whether the decision tree classification is correct or not, and if the number of samples is large enough, the decision tree classification will be more correct.

## 6. Conclusion

In the era of big data, the content of enterprise financial analysis has increased and the complexity of work is higher. The reasonable application of data mining technology can reduce the work pressure of financial personnel and can improve the quality and efficiency of financial analysis, so it is recommended to promote the use. Good foundation to play the role of data support. During the enterprise cost efficiency accounting, data mining technology can be applied to analyze the association of a certain type of cost and another directly unrelated cost. If it has high correlation characteristics, it needs to be integrated into the process of project budgeting and decision-making to improve the accuracy of cost-benefit accounting.

## Figures and Tables

**Table 1:** Table of experimental attributes.

Attribute code	Attribute	Calculation formula
*A*1	Return on assets	(Total profit + finance costs)/(Total assets + total assets of the previous period)^*∗*^2)
*A*2	Gearing ratio	Total liabilities/Total assets
*A*3	Net profit on total assets	Net Income/(Total assets + total assets of the previous period)^*∗*^2
*A*4	Return on assets	Net income/(Total shareholders' equity + total shareholders' equity of the previous period)^*∗*^2
*A*5	Operating income net profit ratio	Profit from main business/income from main business
*A*6	Quick ratio	(Total current assets net inventory) total current liabilities
*A*7	Current ratio	Total current assets/total current liabilities
*A*8	Fixed assets ratio	Total fixed assets/total assets
*A*9	Inventory turnover ratio	Cost of main business/(net inventory + net inventory of previous period)^*∗*^2
*A*10	Interest cover multiplier	(Net profit + income tax + finance costs)/financial costs
*A*11	Total assets turnover ratio	Income from main business/(Total assets + total assets of the previous period)^*∗*^2
*A*12	Working capital to total assets ratio	(Total current assets. Total current liabilities)/Total assets
*A*13	Cash from main business ratio	Cash flow from operating activities/income from main business
*A*14	Accounts receivable turnover ratio	Income from main business (accounts receivable + prior period accounts receivable)^*∗*^2
*A*15	Fixed assets turnover ratio	Revenue from main business/(Total fixed assets + total fixed assets of the previous period)^*∗*^2
*A*16	Accounts receivable turnover ratio	Income from main business (total fixed assets + total fixed assets of the previous period)^*∗*^2
*A*17	Capital adequacy ratio	Total shareholders' equity/Total assets
*A*18	Inventory current liability ratio	Net inventory/Total liquidity liabilities
*A*19	Cash flow to current liabilities ratio	Total cash flow from operating activities/Current liabilities
*A*20	Net income growth rate	Net profit for the period/Net profit for the previous period
*A*21	Operating profit growth rate	Operating profit for the period/Operating profit for the previous period
*A*22	Main revenue growth rate	Income from main business for the period/Income from main business for the period
*A*23	Net assets growth rate	Net assets for the period/Net assets for the previous period
*A*24	Debt capital ratio	Total liabilities/Total shareholders' equity

**Table 2:** Comparison of the correct classification rate of multiple stochastic decisions.

Verification times	Small risk %	Less risky %	Risky %	High risk %
1	87.95	78.58	72.71	54.33
2	88.36	79.23	73.98	53.35
3	89.21	80.03	72.55	45.99
4	88.25	79.65	73.39	58.39
5	86.59	78.54	73.38	52.25
Average	88.01	78.61	73.68	52.63

**Table 3:** Comparison of C4.5 classification accuracy.

Verification times	Small risk %	Less risky %	Risky %	High risk %
1	72.58	65.35	6.31	35.59
2	73.36	66.78	62.19	37.12
3	74.55	66.52	66.37	34.39
4	73.98	65.35	62.98	42.86
5	75.91	66.29	63.28	33.98
Average	74.01	66.22	62.58	36.59

**Table 4:** Comparison of the correct rate of stratified sampling for multiple random decision trees.

Verification times	Small risk %	Less risky %	Risky %	High risk %
1	89.33	86.36	77.55	71.03
2	90.32	85.22	76.53	71.98
3	91.39	88.26	78.96	71.65
4	88.36	86.12	79.32	70.11
5	88.96	88.69	79.89	75.97
Average	88.98	84.98	78.03	71.56

## Data Availability

The experimental data used to support the findings of this study are available from the corresponding author upon request.
